# Associations of family relationships and negative life events with depressive symptoms among Chinese adolescents: A cross-sectional study

**DOI:** 10.1371/journal.pone.0219939

**Published:** 2019-07-18

**Authors:** Zheng Ren, Ge Zhou, Qi Wang, Wenjing Xiong, Juan Ma, Minfu He, Yue Shen, Xinwen Fan, Xia Guo, Ping Gong, Meitian Liu, Xiaodi Yang, Hongjian Liu, Xiumin Zhang

**Affiliations:** 1 Department of Social Medicine and Health Management, School of Public Health, Jilin University, Changchun, China; 2 Department of Epidemiology and Biostatistics, School of Public Health, Jilin University, Changchun, China; 3 China Population Communication Center, Beijing, China; Chiba Daigaku, JAPAN

## Abstract

**Objectives:**

The main objective of this study was to explore the associations of family relationships and negative life events with depressive symptoms among Chinese adolescents.

**Methods:**

A cross-sectional study of 3081 middle school students was conducted in Ganzhou City, Jiangxi Province, China. Students were asked to complete questionnaires regarding family relationships, negative life events, and depressive symptoms. A mediation analysis was carried out using a multiple regression analysis and the PROCESS macro method.

**Results:**

Of all participants, 19.9% reported experiencing depressive symptoms. The prevalence of depressive symptoms was 13.0% and 29.2% in participants with good and poor parental relationships, and the prevalence of depressive symptoms was 11.4% and 30.9% in participants with closed and alienated parental-child relationships, respectively. Parental relationships, parental-child relationships, and negative life events were positively correlated with depressive symptoms. The effect of parental relationships on depressive symptoms was fully mediated by negative life events (Effect = 0.052, 95% CI = [0.023, 0.082]), while the effect of parent-child relationships on adolescent depressive symptoms was partially mediated by negative life events (Effect = 0.075, 95% CI = [0.048, 0.104]).

**Conclusions:**

Our results showed a high prevalence of depressive symptoms in Chinese adolescents. Poor family relationships may have the potential to increase the risk of depressive symptoms, and they could affect depressive symptoms through negative life events.

## Introduction

Adolescence is a critical life transition period marked by substantial physical, behavioral, cognitive, and emotional changes, and it is a period during which adolescents are under considerable social pressure, especially in China [1, 2]. Depression is very common among adolescents worldwide, with a depressive disorder prevalence of approximately 6% [3] and a significant global disease burden [4]. In China, a meta-analysis reported that the pooled point prevalence of major depressive disorders in children and adolescents was 1.3% [5]. Depressive symptoms, though not an adequate standard for the clinical diagnosis of depressive disorders, seem to be quite stable throughout adolescence, and individuals who experience depressive symptoms earlier in adolescence are more likely to continue reporting depressive symptoms later in life [6, 7]. A recent systematic analysis showed that 24.3% of adolescents in secondary schools in mainland China suffered from depressive symptoms [8]. Depressive symptoms can bring about many negative consequences, such as poor academic performance [9, 10], poor interpersonal relationships [11], and antisocial behaviors [12]. Moreover, depression can result in suicide [13].

Understanding the risk factors of depressive symptoms is a vital first step in developing effective preventive strategies and measures. Studies on depressive symptoms in adolescents have reported various characteristics or associated factors, including sex [14, 15], grade [16], health status [17], personality vulnerabilities (e.g. low self-esteem) [18, 19], and school environment (such as student-student and student-teacher relationships, classroom management practices) [20, 21]. The transition to adolescence is indicated not only by the development of a reproductively mature body but also by a plethora of challenges in almost every domain of life. Various studies have reported that during this transition into adult life, some young people experience stressful events that, depending on their type and intensity, may lead to psychological problems of an externalizing (aggressiveness and antisocial behavior) and/or internalizing nature (depression and anxiety) [22, 23]. It has been found that the presence of negative life events is a reliable risk factor for the development of depressive symptoms [24, 25]. Adolescence is also characterized by substantial room for change in attachment relationships. Adolescents face important interpersonal challenges, including the renegotiation of relationships with parents and increased involvement with peers and friends [26]. Family is the most stable source of support throughout adolescence. A growing body of evidence highlights the important role of families in the prevention of internalizing problems in children and adolescents [27–29], of which depressive symptoms are the most frequently reported to be associated with family relationships among adolescents. Factors impacting depressive symptoms include parental warmth [30], support from parents [31] or inter-parental conflict, parental divorce [32, 33], and poor parent-child relationships quality [34, 35]. In fact, the relationship between family cohesion and depressive symptoms has been found in longitudinal work as well, such that decreased perceived family cohesion predicted increased depressive symptoms 1 year later in a study of adolescents [36]. The family systems theory [37] considers that family, as a basic emotional unit, has influence over maintaining the homeostasis of the system. The system may be easy to disrupt if parents have conflicts with each other. When children perceive instability of the system brought about by parental marital conflict, they may engage in problematic behavior. Family problems could lead to insecurity, conflicts with classmates and other negative problems in adolescents. In a two-year longitudinal study, the combined effects of an unfavorable parent-adolescent attachment with negative life events on mental health was found to be larger than the sum of the two individual effects [38]. However, few studies have examined the role of negative life events in family relationships and depressive symptoms.

Given that negative life events are well established as a risk factor for depressive symptoms, we hypothesize that family relationships (parental relationships and parent-child relationships) are related to depressive symptoms among adolescents and that this relationship is mediated by negative life events. Therefore, the aims of the current study were to (1) describe the prevalence of depressive symptoms among middle school students with different types of family relationships; (2) explore the associations of family relationships and negative life events with depressive symptoms in a large sample of adolescents 11–16 years of age in China; and (3) clarify the role of negative life events between family relationships and depressive symptoms.

## Methods

### Participants

This cross-sectional study was conducted in Ganzhou City, Jiangxi Province, China, from September to October in 2017. Four regions were selected for this study: two in rural areas and two in cities. Two schools were chosen at each survey point. In total, eight schools were selected, and three classes were randomly selected from each grade in each school. There were 72 classes in the selected schools included in the study. The well-trained data collectors distributed the paper questionnaires to participants, and the participants completed questionnaires in the classroom during regular class time. A total of 3176 students participated in the questionnaire survey. Of the 3176 students, the data from 95 students were excluded due to missing data, and the final sample size for the present analyses was 3081. This study received approval from the Ethics Committee of Jilin University School of Public Health (No. 2017-08-16). The goals of the study were shared with all of the participants and they expressed their willingness to participate. Written informed consent was obtained from participants and their parents or legal guardians. The characteristics of the participants are reported in Tables 1 and 2.

**Table 1:** Sample basic characteristics according to the categorized parental relationships (Ganzhou, China).

Variables		Sample (n = 3081)	Parental relationships		OR
			Good (n = 1765)	Poor (n = 1316)	
Sex, n (%)	Male	1565	873(49.5)	692(52.6)	1
	Female	1516	892(50.5)	624(47.4)	0.883 (0.765,1.018)
Grade, n (%)***	First	979	607(34.4)	372(28.3)	1
	Second	1085	620(35.1)	465(35.3)	1.224 (1.026,1.460)
	Third	1017	538(30.5)	479(36.4)	1.453 (1.215,1.736)
School type, n (%)***	Urban	1552	955(54.1)	597(45.4)	1
	Rural	1529	810(45.9)	719(54.6)	1.420 (1.230,1.639)
Smoking, n (%)***	No	3003	1743(98.8)	1260(95.7)	1
	Yes	78	22(1.2)	56(4.3)	3.521 (2.139,5.797)
Drinking alcohol, n (%)***	No	2086	1278(72.4)	808(61.4)	1
	Yes	995	487(27.6)	508(38.6)	1.650 (1.417,1.921)
Physical exercise, n (%)***	Lack of exercise	2251	1238(70.1)	1013(77.0)	1
	Often exercise	830	527(29.9)	303(23.0)	0.703 (0.596,0.828)
Self-rated health, n (%)***	Good	1724	1128(63.9)	596(45.3)	1
	Fair	1168	564(32.0)	604(45.9)	3.007 (2.208,4.097)
	Poor	189	73(4.1)	116(8.8)	2.027 (1.742,2.359)
Self-perceived life stress, n (%)***	Low	941	674(38.2)	267(20.3)	1
	Fair	1690	901(51.0)	789(60.0)	2.211 (1.863,2.623)
	High	450	190(10.8)	260(19.7)	3.454 (2.732,4.368)
Self-perceived study stress, n (%)***	Low	536	390(22.1)	146(11.1)	1
	Fair	1554	881(49.9)	673(51.1)	2.041 (1.646,2.530)
	High	991	494(28.0)	497(37.8)	2.687 (2.141,3.373)
Residential area, n (%)***	Urban	1496	935(53.0)	561(42.6)	1
	Rural	1585	830(47.0)	557(57.4)	1.516 (1.313,1.750)
Family type, n (%)***	Stem family	1060	675(38.2)	385(29.3)	1
	Nuclear family	1669	1074(60.8)	595(45.2)	0.971 (0.827,1.140)
	Single parent family	271	16(0.9)	255(19.4)	27.942 (16.606,47.019)
	Foster family	81	0(0.0)	81(6.2)	—
Single child in the family, n (%)	No	2684	1535(87.0)	1149(87.3)	1
	Yes	397	230(13.0)	167(12.7)	1.031 (0.833,1.276)
Fathers’ education level, n (%)***	Junior college or greater	334	231(13.1)	103(7.8)	1
	Senior school	671	423(24.0)	248(18.9)	1.315 (0.994,1.740)
	Junior middle school	1647	903(51.1)	744(56.5)	1.848 (1.437,2,377)
	Primary school or less	429	208(11.8)	221(16.8)	2.383 (1.766,3.215)
Mothers’ education level, n (%)***	Junior college or greater	252	176(10.0)	76(5.8)	1
	Senior school	464	313(17.7)	151(11.5)	1.117 (0.802,1.557)
	Junior middle school	1447	835(47.3)	612(46.5)	1.697 (1.272,2.265)
	Primary school or less	918	441(25.0)	477(36.2)	2.505 (1.858,3.376)
Parent-child relationships, n (%)***	Closeness	1740	1387(78.6)	353(26.8)	1
	Alienation	1341	378(21.4)	963(73.2)	10.010 (8.473,11.826)
Life events***	`X±S		48.25±17.81	57.42±20.07	1.026 (1.022,1.030)

Values are shown as the number (proportions) for categorical data and the mean±SD for continuous data. *P*-values were calculated by *t*-test for continuous variable and *χ*^*2*^-tests for categorical variables. ORs, abbreviation of odds ratios, were derived in logistic regressions modeling the probability of having poor parental relationships.

*** *P* < 0.001.

**Table 2:** Sample basic characteristics according to the categorized parent-child relationships (Ganzhou, China).

Variables		Sample (n = 3081)	Parent-child relationships		OR
			Closeness (n = 1740)	Alienation (n = 1341)	
Sex, n (%)	Male	1565	877(50.4)	688(51.3)	1
	Female	1516	86349.6)	653(48.7)	0.965 (0.836,1.112)
Grade, n (%)***	First	979	639(36.7)	340(25.4)	1
	Second	1085	593(34.1)	492(36.6)	1.559 (1.305,1.863)
	Third	1017	508(29.2)	509(38.0)	1.883 (1.573,2.255)
School type, n (%)***	Urban	1552	943(54.2)	609(45.4)	1
	Rural	1529	797(45.8)	732(54.6)	1.422 (1.233,1.641)
Smoking, n (%)***	No	3003	1714(98.5)	1289(96.1)	1
	Yes	78	26(1.5)	52(3.9)	2.659 (1.652,4.282)
Drinking alcohol, n (%)***	No	2086	1276(73.3)	810(60.4)	1
	Yes	995	464(26.7)	531(39.6)	1.803 (1.548,2.100)
Physical exercise, n (%)***	Lack of exercise	2251	1225(70.4)	1026(76.5)	1
	Often exercise	830	515(29.6)	315(23.5)	0.730 (0.621,0.859)
Self-rated health, n (%)***	Good	1724	1115(64.0)	609(45.4)	1
	Fair	1168	549(31.6)	619(46.2)	2.722 (2.002,3.701)
	Poor	189	76(4.4)	113(8.4)	2.064 (1.774,2.402)
Self-perceived life stress, n (%)***	Low	941	670(38.5)	271(20.2)	1
	Fair	1690	906(52.1)	784(58.5)	2.139 (1.804,2.537)
	High	450	164(9.4)	286(21.3)	4.311 (3.397,5.471)
Self-perceived study stress, n (%)***	Low	536	389(22.4)	147(11.0)	1
	Fair	1554	880(50.5)	674(50.3)	2.027 (1.635,2.512)
	High	991	471(27.1)	520(38.7)	2.922 (2.328,3.666)
Residential area, n (%)***	Urban	1496	930(53.4)	566(42.2)	1
	Rural	1585	810(46.6)	775(57.8)	1.572 (1.362,1.815)
Family type, n (%)***	Stem family	1060	598(34.4)	462(34.4)	1
	Nuclear family	1669	999(57.4)	670(50.0)	0.868 (0.743,1.105)
	Single parent family	271	111(6.4)	160(11.9)	1.866 (1.423,2,446)
	Foster family	81	32(1.8)	49(3.7)	1.982 (1.249,3.145)
Single child in the family, n (%)*	No	2684	1489(85.6)	1195(89.1)	1
	Yes	397	251(14.4)	146(10.9)	1.380 (1.110,1.715)
Fathers’ education level, n (%)***	Junior college or greater	334	228(13.2)	106(7.9)	1
	Senior school	671	420(24.1)	251(18.7)	1.285 (0.973,1.698)
	Junior middle school	1647	883(50.7)	764(57.0)	1.861 (1.449,2,389)
	Primary school or less	429	209(12.0)	220(16.4)	2.264 (1.680,3.051)
Mothers’ education level, n (%)***	Junior college or greater	252	161(9.3)	91(6.8)	1
	Senior school	464	312(17.9)	152(11.3)	0.862 (0.625,1,189)
	Junior middle school	1447	801(46.0)	646(48.2)	1.427 (1.081,1.883)
	Primary school or less	918	466(26.8)	452(33.7)	1.716 (1.287,2.288)
Parental relationships, n (%)***	Good	1765	1387(79.7)	378(28.2)	1
	Poor	1316	353(20.3)	963(71.8)	10.010 (8.473,11.826)
Life events***	`X±S		47.74±17.18	57.91±20.46	1.019 (1.025,1.034)

Values are shown as the number and proportions for categorical data and the mean±SD for continuous data. *P*-values were calculated by *t*-test for continuous variable and *χ*^*2*^-tests for categorical variables. ORs, abbreviation of odds ratios, were derived in logistic regressions modeling the probability of having alienated parent-child relationships.

* *P* < 0.05.

*** *P* < 0.001.

### Students’ information

The information was obtained using one self-administered questionnaire created by the research team. The questionnaire comprised information about individual characteristics: sex, grade, school type (urban or rural); and health-related behaviors, namely, tobacco and alcohol consumption, as well as physical activity. Concerning tobacco and alcohol, participants were classified as nonsmokers/nondrinkers if they had never used and as smokers/drinkers if they smoke/drank regardless of the frequency and quantity [39]. Regarding exercise status, participants were asked how often did they usually exercise per week outside school hours, and the frequency of physical exercise was divided into two categories: frequent exercise (greater or equal to three times per week) and lack of exercise (less than three times per week) [40]. Self-rated health was assessed by asking participants what they thought of their health status in the previous year, with the following response options: very poor, poor, fair, good and very good. In addition, life stress and study stress were evaluated with the following two questions: 1) How do you feel about your life stress in the previous years? and 2) How do you feel about your study stress in the previous years? The response options for the two questions included very low, low, fair, high and very high. Due to the small number of responses in some categories, we combined the data on self-rated health status and self-rated life and study stress into three categories (self-rated health: poor, fair and good; self-rated life or study stress: low, fair and high).

### Family information

Participants provided details about their family and included the following information: residential background (urban or rural), family type (stem, nuclear, single parent or foster family), and parents’ education (junior college or greater, senior school, junior middle school, or primary school or less). Stem family is defined as a family model in which parents and a married child live together. It usually includes immediate family members such as grandparents, parents and unmarried children. The stem family is the transition from extended family to nuclear family. Nuclear family, also known as small family, is a family unit based on marriage. It usually includes husband, wife and their unmarried children. Single-parent family refers to a family consisting of one parent and children. Foster family refers to parents who have children in relatives’ homes due to work or other reasons. The parental relationship was assessed with a single question: How is your parental relationships? Response options included good and poor [41]. The parent-child relationship was evaluated with the following two questions: 1) What is your father's relationship with you? and 2) What is your mother's relationship with you? The response options included closeness and alienation [42]. If the participant did not have parents, the corresponding option would have a missing value, so the above process of deleting the missing value included excluding participants without parents.

### Negative life event assessment

The degree of negative-event-induced stress was determined by the Chinese version of the Adolescent Self-Rating Life Events Checklist (ASLEC), a 6-point scale developed by Liu Xianchen and his colleagues [43], who combined the characteristics of Chinese teenagers based on Chinese and foreign literature. The checklist consists of 27 items regarding six factors: interpersonal conflict; academic pressure; punishment; loss; health and adaptation problems and others. If participants had experienced an event, they were asked to rate the extent to which the event influenced them over the last 12 months. Items were evaluated on a scale of 1 (not occurring) to 6 (influenced me extremely). The reliability and validity of the ASLEC have been supported in samples of Chinese college students [44]. The Cronbach’s alpha for ASLEC was 0.928 in the current study.

### Depressive symptom assessment

To assess depressive symptoms, the Chinese Secondary School Students Depression Scale (CSSSDS) developed by Wang Jisheng was utilized [45]. The scale contains 20 items and each item is rated on a 5-point scale ranging from 1–5. The final scores were calculated by the sum of the scores of all items and divided by 20. The scores ranged from 0–5, and the cut-off used to identify participants presenting depressive problems was > 2. The Cronbach’s alpha for CSSSDS was 0.944 in the current study.

### Data analysis

The data are presented as the mean with standard deviation (SD) and number (proportions). The chi-square test for categorical variables and *t*-test for continuous variable were used to assess the differences in basic characteristics according to the categorized parental relationships and parent-child relationships. Because the dependent variable of the depressive symptoms was classified into two categories, logistic regression models were used to analyze the associations among parental relationships, parent-child relationships and depressive symptoms. The odds ratios (ORs) and 95% confidence intervals (CI) were adjusted with the following potentially confounding factors: sex, grade, school type, smoking, drinking alcohol, physical exercise, self-rated health, self-perceived life stress, self-perceived study stress, residential area, family type, single child in the family, fathers’ education, mothers’ education, parent-child/parental relationships, and negative life events. The ORs were calculated as the exponentiated coefficient from logistic models. Pearson correlation analyze were used to analyze the correlation between variables. Multiple linear regression analysis [46] and a PROCESS macro [47] were used to test and reconfirm the mediating effect of negative life events between parental relationships/parent-child relationships and depressive symptoms. We calculated 95% bootstrap CI based on 5000 bootstrapped samples. *P* values were two-sided and values less than 0.05 were considered statistically significant. The statistical analysis was performed with SPSS 24.0 (IBM Corp, Armonk, New York, USA).

## Results

### Sample characteristic and depressive symptoms

The sample comprised 1565 (50.8%) males and 1516 (49.2%) females, with ages ranging from 11 to 16 (Mean = 13.51 years, SD = 1.07). The study included 979 (31.8%) students in grade one, 1085 (35.2%) students in grade two and 1017 (33.0%) students in grade three. Additionally, a total of 1316 (42.7%) participants reported that their parents’ relationships were poor, and 1341 (43.5%) participants reported that their parent-child relationships were alienated.

Table 1 shows the basic characteristics of the participants according to the categorized parental relationships. Among all the participants, those with poor parental relationships reported a higher proportion of smoking, drinking, and lack of physical exercise than those with good parental relationships (*P* < 0.001). Statistical significance was observed for the distribution of all basic characteristics except for the variables of sex and single child in the family among different parental relationships groups. Details of the basic characteristics of participants with different parental relationships are described in Table 1.

Table 2 presents characteristics of the sample across the different categories of parent-child relationships groups. Groups differed on all basic characteristics except sex. The proportions of participants in the third grade with close and alienated parent-child relationships were 29.2% and 38.0%, respectively. Additionally, participants with an alienated parent-child relationship reported a higher proportion of high life and study stress than those with a good parental relationship (*P* < 0.001). Details of the basic characteristics of participants with different categories of parent-child relationships groups are described in Table 2.

The results of the cross-sectional analysis of depressive symptoms in participants with different groups of parental relationships and parent-child relationships are presented in Table 3. Of all participants, 19.9% reported experiencing depressive symptoms. The prevalence of depressive symptoms was 29.2% and 30.9% in poor parental relationships and alienated parent-child relationships, respectively. Moreover, compared with participants with good parental relationships and close parent-child relationships, participants with poor parental relationships and alienated parent-child relationships were 2.750 (OR 2.750, 95% CI = [2.291, 3.301]) and 3.470 (OR 3.470, 95% CI = [2.877, 4.187]) times as likely to have depressive symptoms, respectively. After adjusting for potential confounding factors, the value of the OR decreased to 1.664 (95% CI = [1.272, 2.718]) but was still statistically significant for participants with alienated parent-child relationships. However, for participants with poor parental relationships, no statistical significance was detected (OR 1.223, 95%CI = [0.925, 1.616]) after adjustment.

**Table 3:** Parental relationships and parent-child relationships according to the categorized depressive symptoms (CSSSDS ≥ 2) (Ganzhou, China).

Variables		Sample	(CSSSDS ≥ 2)		Unadjusted OR	Adjusted OR
			n	%		
Parental relationships	Good	1765	230	13.0	1	1
	Poor	1316	384	29.2	2.750 (2.291,3.301)	1.223 (0.925,1.616)^a^
Parent-child relationships	Closeness	1740	199	11.4	1	1
	Alienation	1341	415	30.9	3.470 (2.877,4.187)	1.664 (1.272,2.718)^b^
Overall		3081	614	19.9		

CSSSDS, Chinese Secondary School Students Depression Scale.

Values are shown as numbers for categorical data.

^a^ Results adjusted for sex, grade, school type, smoking, drinking alcohol, physical exercise, self-rated health, self-perceived life stress, self-perceived study stress, residential area, family type, single child in the family, fathers’ education level, mothers’ education level, parent-child relationships, and negative life events.

^b^ Results adjusted for sex, grade, school type, smoking, drinking alcohol, physical exercise, self-rated health, self-perceived life stress, self-perceived study stress, residential area, family type, single child in the family, fathers’ education level, mothers’ education level, parental relationships, and negative life events.

### Analysis of correlations

The pearson correlations (two-tailed) for parental relationships/parent-child relationships, negative life events, and depressive symptoms are presented in Table 4. The results showed that the correlation coefficients ranged from 0.234 to 0.692 and all were significant (*P* < 0.001). parental relationships, parent-child relationships, and negative life events were positively correlated with depressive symptoms. Moreover, parental relationships and parent-child relationships were positively correlated with negative life events.

**Table 4:** Correlations among variables (Ganzhou, China) (n = 3081).

Variables	1	2	3	4
1. Parental relationships	1			
Parent-child relationships	—	1		
Life events	0.234***	0.261***	1	
Depressive symptoms	0.236***	0.289***	0.692***	1

Pearson correlations were used to analyze the associations among parental relationships/parent-child relationships, negative life events and depressive symptoms.

*** *P* < 0.001.

### Testing for the mediation effect

To test the mediating effects, a multiple linear regression analysis was used to examine whether negative life events mediate the association between parental relationships/parent-child relationships and depressive symptoms. Furthermore, we adjusted the confounding factors that were significant to the independent variables in each mediation effect analysis. Table 5 and Table 6 display the testing results. In the first step, parental relationships/parent-child relationships were significantly associated with depressive symptoms (*B* = 0.058, *β* = 0.046, *P* = 0.016 for parental relationships and *B* = 0.163, *β* = 0.130, *P* < 0.001 for parent-child relationships). In the second step, parental relationships/parent-child relationships were significantly associated with negative life events (*B* = 2.837, *β* = 0.073, *P* < 0.001 for parental relationships and *B* = 4.047, *β* = 0.104, *P* < 0.001 for parent-child relationships). In the third step, when we controlled for parental relationships/parent-child relationships respectively, negative life events were associated with depressive symptoms (*B* = 0.018, *β* = 0.573, *P* < 0.001), but the effect of parental relationships on depressive symptoms was disappeared (*B* = 0.005, *β* = 0.004, *P* = 0.784). The effect of parent-child relationships on depressive symptoms was reduced (*B* = 0.088, *β* = 0.070, *P* < 0.001).

**Table 5:** Testing the mediation effect of life events in the association between parental relationships and depressive symptoms (Ganzhou, China) (n = 3081).

Variable	*B*	β	*t*	*R*^*2*^	*F*
Step 1: Parental relationships predicts depressive symptoms					
Independent variable: parental relationships	0.058	0.046	2.407***	0.278	84.394***
Dependent variable: depressive symptoms					
Step 2: Parental relationships predicts negative life events					
Independent variable: parental relationships	2.837	0.073	3.695***	0.231	65.914***
Dependent variable: negative life events					
Step 3: Negative life events predicts depressive symptoms					
Independent variable: parental relationships	0.005	0.004	0.274	0.484	1446.353***
Mediator: negative life events	0.018	0.573	40.621***	0.531	231.135***
Dependent variable: depressive symptoms					

The results adjusted for grade, school type, smoking, drinking alcohol, physical exercise, self-rated health, self-perceived life stress, self-perceived study stress, residential area, family type, fathers’ education level, mothers’ education level, and parent-child relationships.

*** *P* < 0.001.

**Table 6:** Testing the mediation effect of life events in the association between parent-child relationships and depressive symptoms (Ganzhou, China) (n = 3081).

Variable	*B*	β	*t*	*R*^*2*^	*F*
Step 1: Parent-child relationships predicts depressive symptoms					
Independent variable: parent-child relationships	0.163	0.130	7.053***	0.278	78.813***
Dependent variable: depressive symptoms					
Step 2: Parent-child relationships predicts negative life events					
Independent variable: parent-child relationships	4.047	0.104	5.458***	0.231	61.512***
Dependent variable: negative life events					
Step 3: Negative life events predicts depressive symptoms					
Independent variable: parent-child relationships	0.088	0.070	4.719***	0.491	1487.517***
Mediator: negative life events	0.018	0.573	40.635***	0.531	216.871***
Dependent variable: depressive symptoms					

The results adjusted for grade, school type, smoking, drinking alcohol, physical exercise, self-rated health, self-perceived life stress, self-perceived study stress, residential area, family type, single child in the family, fathers’ education level, mothers’ education level, and parental relationships.

*** *P* < 0.001.

In addition, we reconfirmed the effect by employing the PROCESS macro (Model 4) in SPSS to perform the bootstrap method. The results showed that the direct effect of parental relationships on depressive symptoms disappeared and that the direct effect of parent-child relationships on depressive symptoms was reduced. We calculated the 95% CI based on 5000 bootstrap resampling. The indirect effects of parental relationships and parent-child relationships on depressive symptoms through negative life events (95% CI = [0.023, 0.082], 95% CI = [0.048, 0.104]) were both significant, as zero was not contained in the 95% CI. Thus, we confirmed that negative life events fully mediated the association between parental relationships and depressive symptoms and partially mediated the association between parent-child relationships and depressive symptoms.

## Discussion

Our study selected middle school students as the participants and found that poor parental relationships/parent-child relationships were associated with an increased risk of depressive symptoms before adjusting for potential confounders, while poor parental relationships became not statistically significant after the adjustments. This result laid the foundation for subsequent study of the role of negative life events. We formulated a mediation model to examine whether negative life events mediate the association between family relationships and depressive symptoms. Our findings showed that the effect of parental relationships on adolescent depressive symptoms was fully mediated by negative life events, while the effect of parent-child relationships on adolescent depressive symptoms was partly mediated by negative life events. The findings of this study have the potential to extend our knowledge about the relationship between family relationships and depressive symptoms in adolescents, which may be helpful for the improvement of family relationships and the prevention of depressive symptom development in adolescents.

Using the CSSSDS with a cut-off score of 2, our study found that the prevalence of depressive symptoms among middle school students in Ganzhou city was 19.9%, and the result was similar to previous literature findings [16, 39, 48, 49]. We also found that the prevalence of depressive symptoms was 13.0% and 11.4% with good parental relationships and parent-child relationships, and the prevalence increased to 29.6% and 31.1% among those with poor parental relationships and parent-child relationships, respectively. A poor parent-child relationship could increase the risk of depressive symptoms even if adjusted for negative life events and other potentially confounding factors, which supported the findings of previous studies [38, 50]. The influence of family relationships on depressive symptoms can be explained as follows: according to attachment theory [51], theories regarding the connectedness-individuation link [52], and theories dealing with parenting practices [53], positive developmental outcomes with lower levels of depressive symptoms occur when adolescents have close relationships with their parents. Lower-quality relationships with parents may result in more depressive symptoms because adolescents in such relationships gain low support from their parents when facing emotional problems [54]. Despite normative changes in the parent-child relationships during adolescence, including greater strain and conflict, the family remains a critical source of support, and adolescents continue to use their parents as a guide for understanding and determining how to approach emotions.

The correlation analysis of this study showed that parental relationships/parent-child relationships were related to negative life events and depressive symptoms, as confirmed in other studies [55, 56]. Possible explanation of our findings could be that better family relationships are associated with fewer negative life events and fewer depressive symptoms. A harmonious family relationship could lead to resolution of a negative life event by the mutual care and mutual help of family members. This may help them be more positive and less likely to have a mental illness. Our study suggested that negative life events positively predict depressive symptoms and this finding supported that individuals with more negative life events were more likely to suffer from depressive symptoms [57].

We also observed the mediating effect of negative life events between family relationships and depressive symptoms. The effect of parental relationships on adolescent depressive symptoms was fully mediated by negative life events, while the effect of parent-child relationships on adolescent depressive symptoms was partly mediated by negative life events, which confirmed our hypothesis. Simple mediation analyses suggested that adolescents who were frequently exposed to poor family relationships would have more negative feelings about negative life events or even experience more negative life events which, in turn, can lead to depressive symptoms. The present findings were in accordance with separate lines of research showing that 1) family factors can predict adolescent’s depressive symptoms [58, 59], 2) supportive family relationships are linked to negative life events [55], and 3) negative life events are associated with depressive symptoms [25, 60]. According to our mediation models, the effect of negative life events on parental relationships/parent-child relationships and depressive symptoms may be explained as follows.

According to family systems theory, a good parental relationship is the cornerstone of family harmony, and a parent-child triangular relationship seeks to stabilize parental emotion, alleviate family conflicts, and form a balanced family system [37]. Family cohesiveness, a home life characterized by emotional warmth and affection, and emotional support from parents who are accessible and available are known factors that protect against depression [61]. A previous study has pointed out the important role of supportive family relationships in the behavioral adjustment of adolescents, such as those characterized by cohesion, communication and affection, which could protect them against some negative consequences of stressful life events [55], and may not increase the risk of mental health problems. Even if negative life events occur, individuals with good family relationships will receive support from their family members, appraise the stressors properly and adopt positive coping strategies [62]. Accordingly, parent-child communication may influence the degree of depressive symptoms by shaping children’s stress coping abilities.

Conversely, a poor family relationship may have a negative effect on the development of adolescents. Parental divorce was a significant risk factor for depressive symptoms in adolescents [33]. Adolescents were not the main participants when parents were in conflict, but they may have adopted negative coping strategies and triggered a series of negative life events in order to face the disharmonious family environment. When children were alienated from one or both parents, they did not gain care or support from their parents, which may directly or indirectly affect mental health. A poor family relationship, as a kind of negative life event, may lead to more negative events incidences and make children more prone to negative impacts in real-world situations. For example, children and adolescents in homes with high conflict may have difficulties in their own social relationships, and may be prone to developing poor interpersonal skills with others [63]. In addition, children and adolescents who have experienced the breakdown of parental marriage and conflicts will show frequent problematic behaviors and poor academic performance later in life [64]. In other words, when adolescents were in a disharmonious family relationship, the feelings resulting from negative life events and the effects of negative life events were stronger than in those with good family relationships. The occurrence of many negative life events may increase the possibility of depressive symptoms among adolescents.

Our study has several strengths. First, we used the Chinese Secondary School Students Depression Scale (CSSSDS), which was specifically compiled according to the characteristics of Chinese secondary school students to evaluate the depressive symptoms of participants. Second, to improve the reliability of the results, our study adjusted for most of the confounding factors such as grade, school type, smoking, drinking alcohol, physical exercise, self-rated health, self-perceived life stress, self-perceived study stress, residential area, family type, single child in the family, fathers’ education, mothers’ education, and parental relationships/parent-child relationships.

Several limitations of our study must be acknowledged. First, a cross-sectional survey design cannot determine the directionality of the observed relations. Second, all the information in this study stemmed from self-reported questionnaires completed by the participants; therefore, recall bias may exist in the process of information collection. Third, family relationships were evaluated using only two questions, and they were only suitable for the evaluation of the relationships among fathers, mothers and children and did not include relationship evaluations for participants and other family members.

## Conclusions

Our results showed a high prevalence of depressive symptoms in middle school students. The effect of parental relationships on depressive symptoms was fully mediated by negative life events, and the effect of parent-child relationships on depressive symptoms was partly mediated by negative life events. Much attention should be paid to the relationship between family relationships and negative life events and the development of family-based preventive interventions related to adolescent depressive symptoms.

## Supporting information

S1 Database The database for this study. (SAV)
